# Relatos de exploradores e viajantes e primeiras pesquisas científicas com a ayahuasca, 1850-1950, no debate atual sobre o “renascimento psicodélico”

**DOI:** 10.1590/S0104-59702023000100023

**Published:** 2023-07-10

**Authors:** Vinícius Maurício de Lima, Maria Gabriela Silva Martins da Cunha Marinho

**Affiliations:** i Programa de Pós-graduação em Ciências Humanas e Sociais Universidade Federal do ABC São Bernardo do Campo SP Brasil vmlima9@gmail.com Doutorando, Programa de Pós-graduação em Ciências Humanas e Sociais/Universidade Federal do ABC. São Bernardo do Campo – SP – Brasil vmlima9@gmail.com; ii Programa de Pós-graduação em Ciências Humanas e Sociais Universidade Federal do ABC Professora e pesquisadora, Programa de Pós-graduação em Ciências Humanas e Sociais/Universidade Federal do ABC; pesquisadora associada, Museu Histórico/Faculdade de Medicina/Universidade de São Paulo. São Bernardo do Campo – SP – Brasil gabriela.marinho@ufabc.edu.br; Museu Histórico Faculdade de Medicina Universidade de São Paulo São Bernardo do Campo SP Brasil gabriela.marinho@ufabc.edu.br

**Keywords:** ayahuasca, história das ciências, história indígena, renascimento psicodélico, drogas

## Abstract

O artigo descreve associações e controvérsias entre usos indígenas e ocidentais da ayahuasca, de 1850 a 1950, na relação com o “renascimento psicodélico”. Destaque na ciência desde 2000, esse movimento faz referência a 1960-1970, quando políticas antidrogas suspenderam pesquisas sobre “potenciais terapêuticos” de substâncias psicoativas. Argumenta-se que estudos pioneiros com a ayahuasca datam do início do século XX e mencionam relatos de expedições à Amazônia desde 1850. Esses artigos e relatos são analisados pelo aspecto histórico da teoria do ator-rede e de estudos recentes. Infere-se que a história ilumina o debate político atual sobre os usos, classificações e significados indígenas; o interesse farmacêutico na ayahuasca; e a discussão sobre “drogas”.

A ayahuasca^1^ é beberagem utilizada há milênios por indígenas das terras altas e das terras baixas da Amazônia (atuais territórios de Bolívia, Brasil, Colômbia, Equador, Peru e Venezuela). Estima-se que, atualmente, mais de cem nações indígenas façam seu uso ritual (Assis, Rodrigues, 2017; Tukano, 14 fev. 2019). A partir da segunda metade do século XX, ela vem sendo popularizada no Brasil e no exterior com o advento e a expansão das religiões ayahuasqueiras brasileiras e dos grupos neoayahuasqueiros, que se distinguem daquelas por ser “universalistas” ou por procurar se desvincular de preceitos religiosos ( Labate, 2000 ; Lima, 2021 ). Na esteira das políticas antidrogas dos anos 1960 e 1970, aconteceu, ao longo de décadas, uma perseguição de agentes do Estado, de outras instituições religiosas e de veículos de imprensa às instituições e pessoas que faziam uso religioso da beberagem ( Antunes, 2012 ). Também houve um hiato nas pesquisas sobre os “potenciais terapêuticos” da ayahuasca e de seus compostos químicos, bem como de outras substâncias psicoativas. Somente nos anos 1990 alguns estudos retomaram a discussão ( Grob et al., 2012 ; Strassman, 2019 ).

É, porém, a regulamentação dos usos religiosos e dos usos científicos da ayahuasca em países como os EUA e o Brasil, nas décadas de 2000 e de 2010, que reconhece os usos indígenas e os usos religiosos da beberagem e marca o “renascimento” da “ciência psicodélica”, em áreas como a psiquiatria e a neurociência. Em especial, devido ao crescimento dos investimentos, do número de estudos clínicos e de eventos internacionais sobre usos terapêuticos da ayahuasca e outros “psicodélicos” ( Antunes, 2012 ; Beserra, Vieira, 2020; Guimarães, 19 jul. 2017; Leite, 21 dez. 2020; Rodrigues, Beserra, 2020). Ao tratar desse renascimento, os pesquisadores remetem aos estudos que vinham sendo realizados nas décadas de 1960 e 1970 e que foram suspensos devido às políticas proibicionistas.

Essa menção aos anos 1960 e 1970 nos fez questionar se as primeiras pesquisas sobre os potenciais terapêuticos da ayahuasca são desse momento histórico ou de antes. Com este artigo quisemos demonstrar que, embora o termo “psicodélico” tenha sido criado pelo psiquiatra inglês Humphry Osmond, em cartas que trocava com o escritor também inglês Aldous Huxley, nos anos 1950, e tenha se difundido durante os anos 1960 e 1970 na ciência e pelo movimento de contracultura, demonstrando a relevância do tema naquele período ( Delmanto, 2020 ), foi nos anos 1930 e 1940 que se iniciaram os estudos sobre os potenciais terapêuticos da ayahuasca – naquela época mais conhecida como yagê e caapi. Para desenvolver seus estudos, pesquisadores buscavam então informações nos relatos de exploradores e de viajantes que haviam realizado expedições à Amazônia na segunda metade do século XIX.

Rituais indígenas como o Dabucuri, que utiliza o caapi – ou gaapi, como veremos –, eram mencionados em diversos relatos, como no do naturalista britânico Alfred Russel Wallace, autor, com Charles Darwin, da teoria da evolução das espécies. Essas informações serviram de referência para o desenvolvimento de estudos por botânicos, farmacêuticos e até psiquiatras eugenistas interessados em suas “aplicações”. A despeito dos interesses controversos, nessas pesquisas surgem indícios de que essa beberagem pudesse ser útil no tratamento de problemas de saúde pública como a ansiedade e a depressão, como mostra a literatura atual ( Ona et al., 2019 ; Rocha et al., 2019 ).

Mesmo assim, pensando na linha de reflexão do filósofo brasileiro Pedro Paulo Pimenta (2020) sobre a relação entre humanos e plantas no Ocidente, as primeiras pesquisas subtraíam os usos, as classificações e os significados indígenas da ayahuasca, reduzindo-os a um quadro epistemológico com base nas ciências naturais e em um único conceito de humanidade – o moderno –, por meio da difusão dos usos terapêuticos. Nem mesmo os estudos etnográficos, realizados na primeira metade do século XX, detalhavam aspectos culturais indígenas, como observou o antropólogo austro-colombiano Gerardo Reichel-Dolmatoff (1976) . Afinal, não abordavam os significados das plantas que compõem a ayahuasca e, em última instância, da própria ayahuasca, como têm mostrado estudos mais recentes de pesquisadores indígenas que os colocam, por exemplo, como fundamentais na constituição de sujeitos indígenas (Diakuru, Kisibi, 2006; Fernandes, 2018 ).

O primeiro recenseamento sobre “toxicômanos” realizado em todas as regiões do Brasil, em 1962, diz que a ayahuasca não constituía “um problema médico-social de importância”, como eram consideradas outras substâncias psicoativas como a *Cannabis* spp. ( Parreiras, 1965 , p.44). Porém, os relatos de exploradores e de viajantes e as pesquisas do início do século XX revelam, nos termos do filósofo, sociólogo e antropólogo francês Bruno Latour (2012) , uma “rede” diversificada de associações e controvérsias científicas e sociais sobre os usos indígenas e os usos ocidentais da “planta telepática” que chegava ao Rio de Janeiro, então capital brasileira. Pesquisadores faziam questionamentos morais e jurídicos sobre a beberagem de origem indígena que podia “perverter” os ocidentais, propagando preconceitos sobre os usos da ayahuasca por indígenas e por ocidentais que chegam até nossos dias.

Nesse sentido, nosso objetivo neste artigo é descrever as associações e controvérsias entre os usos indígenas e os usos ocidentais da ayahuasca, de 1850 a 1950, e como se relacionam com o renascimento psicodélico. Para isso, investigamos relatos de exploradores e de viajantes da segunda metade do século XIX, em especial um relato de Wallace (2004) , publicado originalmente em 1889, na obra *Viagens pelo Amazonas e rio Negro* , e quatro artigos científicos publicados em três revistas científicas no Brasil, na primeira metade do século XX, disponibilizados pelo Centro Brasileiro de Informações sobre Drogas Psicotrópicas, da Universidade Federal de São Paulo; além disso, trouxemos literatura contemporânea para discussão.

Partindo da leitura dos relatos de exploradores e de viajantes e dos artigos de pesquisadores, propusemos uma análise do aspecto histórico da teoria do ator-rede, de Latour (2012) , mais difundida nas ciências sociais, mas que, segundo procuramos demonstrar, pode ser utilizada pela história e na historiografia. Para isso, consideramos três tópicos da teoria para análise. No primeiro, pudemos entender que indígenas, exploradores, viajantes e pesquisadores do final do século XIX e início do XX estão, nos termos latourianos, em uma mesma “rede” que indígenas, pesquisadores e outros atores de hoje e estabelecem associações e controvérsias que nos mostram usos, classificações e significados da ayahuasca ao longo de anos.

No segundo tópico, observamos o desdobramento de algumas “camadas” ( Latour, 2020 ), que são acontecimentos pelos quais desvelamos que, antes, pesquisadores relacionavam a ayahuasca apenas ao cipó *Banisteriopsis caapi* . Diferente, portanto, da ayahuasca descrita a partir dos usos religiosos pelas religiões ayahuasqueiras, que realizam a decocção do cipó com as folhas da árvore *Psychotria viridis* , planta sobre a qual estão concentrados os estudos em curso sobre os potenciais de suas propriedades químicas. Entre esses acontecimentos, portanto, estão os relatos de exploradores e de viajantes sobre os usos indígenas, também relatos de antropólogos indígenas de hoje, bem como estudos do início do século XX e de agora, que podem indicar a necessidade de novas pesquisas sobre os potenciais terapêuticos do cipó.

Por fim, no terceiro tópico, ao identificar diferentes temporalidades, demonstramos que elementos pré-modernos e modernos coexistem no passado e nos dias atuais, pois a ayahuasca os congrega como um ator da própria rede, ou, ainda, um “híbrido”, conforme Latour (1994) , e oferece elementos para a discussão política ( Latour, 2012 ) necessária ao renascimento psicodélico, por exemplo, sobre a criminalização de rituais indígenas como o Dabucuri; sobre o enfrentamento de um quadro epistemológico que subtrai os usos, classificações e significados indígenas; sobre o interesse das indústrias farmacêuticas na beberagem e em suas propriedades químicas; sobre os usos terapêuticos da ayahuasca no controle de corpos e mentes; e no debate sobre as “drogas”.

## Relatos de usos e a criminalização de rituais indígenas

Há séculos, exploradores, viajantes e pesquisadores de áreas como botânica, química, farmacologia e etnologia, vindos em especial da Europa, interessam-se pelos usos que indígenas da América do Sul fazem de determinadas plantas, colocando todos esses atores em uma mesma rede na qual estabelecem ligações relativamente estáveis que indicam os motivos pelos quais se unem ou as contradições pelas quais se distanciam ( Latour, 2012 ). Em *O uso das plantas silvestres da América do Sul tropical* , o antropólogo belga Claude Lévi-Strauss (1986 , p.44) faz um apanhado de diversos conhecimentos sobre algumas das plantas utilizadas pelos indígenas, por exemplo, para confecção de bálsamos e remédios, e diz que poucos povos “primitivos” desenvolveram “um conhecimento tão completo sobre as propriedades físicas e químicas de seu ambiente botânico quanto os índios sul-americanos”.

No entanto, quanto às plantas com componentes psicoativos, na Europa havia pouco interesse científico em suas propriedades, o que passa a mudar, em especial, a partir de 1890 com a descoberta da mescalina, composto químico do peyote, um cacto encontrado nas Américas. Mas o interesse científico na mescalina ficou restrito, e foram publicados poucos artigos até os anos 1930 ( Strassman, 2019 ).

Sobre a ayahuasca, os primeiros relatos missionários dos usos indígenas da beberagem na região amazônica aparecem em textos de dois jesuítas, no final do século XVII e início do século XVIII. O espanhol José Chantre y Herrera falou de um “brebaje diabólico”,^2^ e o suíço Juan Magnin disse ter conhecido uma medicina indígena. Também no século XVIII, o jesuíta Pablo Maroni informou sobre uma “bebida intoxicante”, da mesma maneira que o jesuíta Franz Xaver Veigl descreveu o uso da beberagem. Já os primeiros relatos de viajantes datam do início do século XIX, quando o explorador e naturalista suíço Johann Jakob von Tschudi encontrou com indígenas que utilizavam a ayahuasca no Peru ( Schultes, 1986 ). Apesar de comentários breves, foram os relatos desses e de outros exploradores e viajantes que se popularizaram na literatura acadêmica sobre os potenciais terapêuticos da beberagem no início do século XX.

Entre os relatos de viajantes do século XIX, Antunes (2011) destaca os do médico e naturalista britânico Richard Spruce como “caso paradigmático”, devido a ser citado amplamente em trabalhos que vieram na sequência. Spruce viajou 15 anos pela Amazônia, no século XIX, passou um tempo entre povos como os guajibos, os záparos e os tukanos e teria sido o primeiro a colher, no início dos anos 1850,^3^ o caapi e enviar para estudos em herbários da Europa. Suas observações foram publicizadas apenas em 1873 e chegaram ao público em 1908, com a publicação de seu livro *Notes of a botanist on the Amazon and Andes* , em dois volumes, editado por Wallace (Costa, Faria, 1936; Schultes, 1986 ).

Nem sempre, no entanto, esses relatos continham densidade etnográfica, com frequência deturpando os usos, as classificações e os significados indígenas da beberagem. Diziam, por exemplo, que indígenas como os tarianas, os záparos e os shuaras (estes chamados pelos ocidentais genericamente de jíbaros) usavam “alucinógenos”/“alucinatórios” ou “venenos” de efeito “tóxico” como “remédios”, pelos quais também podiam sonhar e tinham práticas de telepatia e clarividência. Spruce, por exemplo, lembra do período em que esteve no Alto Rio Negro e faz menção ao ritual Dabucuri, realizado por diversas nações indígenas que viviam e ainda vivem à noroeste da Amazônia, às margens do rio Uaupés, mas não aprofunda suas análises (Costa, Faria, 1936).

O Dabucuri, da língua geral ou nheengatu, é um ritual milenar de povos indígenas do Alto Rio Negro que agrega um conjunto de conhecimentos, como narrativas da criação da humanidade, ancestralidade e origens das roças. Nele também realizam ritos de passagem, alianças políticas e arranjos matrimoniais. Entre danças, cantos e sons de instrumentos como flautas, uma comunidade de um mesmo clã ou nação oferta, a depender da época do ano, uma grande quantidade de frutos, peixes ou objetos (cestos, balaios e tipitis) e é recebida por outro grupo com a distribuição de caxiri, bebida feita a base de mandioca fermentada consumida à vontade no ritual, de caapi e de tabaco. Posteriormente, esperam a retribuição. Hoje, 23 grupos étnicos de quatro famílias linguísticas (aruak, maku, tukano e yanomami) o praticam pontualmente, cada qual a seu modo (Diakuru, Kisibi, 2006; Waikon, 2016 ).

Os relatos sobre essa cerimônia eram incorporados por pesquisadores, sendo também uma maneira de expressão do etnocentrismo. Embora sejam relevantes os relatos de Spruce, que chega a realizar uma viagem à Amazônia com Wallace, também são interessantes os que o autor da teoria da evolução das espécies conta em seu livro *Viagens pelo Amazonas e rio Negro* , de 1889, quando esteve pela primeira vez no rio Uaupés junto com um branco interlocutor (que nesse relato não era Spruce) e três indígenas. O explorador e naturalista britânico levou objetos ocidentais para trocar com os indígenas por animais, plantas e outros objetos e conta ter ficado regozijado ao encontrar os “legítimos representantes da floresta”, povos de “raças semicivilizadas” ( Wallace, 2004 ).

Para atender a um pedido de Wallace, o tuxaua Calixto, líder indígena de um dos povos que conheceram, determina que seja realizado o ritual em que os indígenas bebem caxiri e o caapi. O explorador e naturalista descreve que cerca de trezentas pessoas, entre homens e mulheres adultos, rapazes, moças e crianças participaram, pintando e adornando com penas os corpos e preparando o ritual. As mulheres produziram o caxiri, com água que buscaram no rio e com líquido da mandioca colhida na roça ao redor das malocas, e pegaram galhos secos na floresta para acender as fogueiras. Enquanto isso os homens trançaram coroas de palha e confeccionaram outros objetos.

Durante a cerimônia, iniciada ao anoitecer, ao som de tambores e flautas, rapazes e moças dançaram e beberam o caxiri na maloca principal. Foi quando começou o que Wallace chamou de “dança da cobra”, parte de um ritual de iniciação de rapazes no qual cada um levava duas cobras enormes feitas com caules de imbaúba. Eles dançaram na porta da maloca com as cobras sobre os ombros, imitando os movimentos do réptil, até entrarem. Então, dentro da maloca as cobras lutaram entre si, enquanto o caxiri era distribuído por outras pessoas aos presentes. Na sequência, os rapazes deixaram as cobras e passaram a distribuir o caxiri.

Depois, foi introduzido o caapi, que era distribuído por um indígena mais velho, no meio da maloca, a partir de um pote grande de barro com duas cuias pequenas. Rapazes com arcos, flechas e lanças vinham aos pares receber a bebida “excessivamente amarga”. Apesar de deixar claro que bebeu o caxiri e gostou, Wallace não confirma se bebeu o caapi. Além disso, embora demonstrasse interesse em sua narrativa por boa parte das plantas que encontrava, Wallace não se detém em entender as relações que esses povos estabelecem com cada uma, tampouco descreve as espécies de plantas utilizadas, a preparação e os significados dos usos do caapi.

A rede, contudo, não fica restrita a pesquisadores e a indígenas do passado e encontra com uma geração de indígenas pesquisadores do presente, atores importantes no campo de estudos sobre a ayahuasca hoje ( Latour, 2012 ), e com uma discussão atual sobre o “saber localizado”, para a qual a variedade de posicionamentos é uma alternativa ao relativismo e denota como os saberes são sempre parciais e localizáveis. Isto é, ao ser definidas situacionalmente, essas conexões possibilitam pensar em “redes de posicionamentos diferenciais”, nas quais o eu é ressignificado quando se assemelha ao outro, neste caso, pelo desenvolvimento de pesquisas por sujeitos com distintas cosmovisões ( Haraway, 1995 ).

Assim, indígenas da etnia desana, também do rio Uaupés, detalham que o Dabucuri é um ritual que envolve toda a comunidade, que recebe os indígenas visitantes para celebrar, em determinados períodos do ano, a terra, os rios, as árvores, os animais, entre outros entes, e suas inter-relações com seus parentes e/ou cunhados, seus antepassados, com outras gentes (como a gente-peixe, a gente-planta etc.) e, em especial, celebram o advento da humanidade, que contam como surgiu por meio do mito de criação da divindade Gaapi. É do Gaapi que os indígenas descendem e é com ele que se ligam por intermédio da planta gaapi, metonímia para a divindade. Esses conhecimentos são transmitidos com zelo para as novas gerações, que dão continuidade à “gente-gaapi” (Diakuru, Kisibi, 2006).

Em seu relato autoetnográfico, o antropólogo indígena brasileiro Jaime Moura Fernandes (2018) explica que, entre os desana, há três tipos de gaapi: o gaapi filho do dia, utilizado em rituais de passagem com jovens de ambos os gêneros; o gaapi de frutas, consumido durante os Dabucuri; e o gaapi de *Waimahsã* , consumido pelos *kumuã* (especialistas em cura) durante os Dabucuri de peixe, estes dois últimos gaapi não são cultivados pelos humanos, pois existem “espontaneamente” na floresta. Ele menciona também o gaapi dos especialistas, utilizados antes das grandes enchentes, portanto, não mais existentes, para “agenciar a vida” e para prever perigos e prevenir doenças. No entanto, não informa se cada gaapi é produzido a partir de uma única espécie morfofisiológica, somente diz que o gaapi é preparado com pat ou ipadu ( *Erythroxylum novogranatense* ), uma árvore da família das eritroxiláceas, cujas folhas são semelhantes às da coca: “Quando o gaapi não é misturado ele não produz o efeito esperado” ( Fernandes, 2018 , p.57).

Mais que uma planta alucinógena, portanto, o gaapi é a divindade da qual os desanas descendem. Assim, enquanto Wallace (2004) relaciona o caapi a uma animosidade entre os indígenas que o bebiam e teriam ficado mais “exaltados”, correndo “furiosamente” pela maloca, como se fossem matar um inimigo, denotando “selvageria”, Fernandes (2018) coloca que os indígenas agem ritualmente com “violência” ao ingerir a beberagem porque, durante o acontecimento, o gaapi liga a gente-gaapi do passado e do presente.

Na primeira perspectiva, as plantas são colocadas como um reino secundário, conforme a história natural e a biologia ocidentais as classificam em relação ao reino animal e, em última instância, à espécie *Homo sapiens* ( Pimenta, 2020 ), que faz uso das plantas para se “entorpecer”. Na segunda perspectiva, as plantas são decisivas na vida dos humanos, como é também, por exemplo, o timbó para os suruwahas, que têm seus modos de existência e morte imbricados com essa leguminosa com propriedades tóxicas ( Aparicio, 2019 ).

Nas pesquisas da primeira metade do século XX, a perspectiva ocidental sobre a ayahuasca não é superada nem mesmo por um etnólogo, o teuto-brasileiro Herbert Baldus, que em 1950 escreve o artigo “Bebidas e narcóticos dos índios do Brasil: sugestões para pesquisas etnográficas”, para a revista *Sociologia* , no qual afirma, ao se referir aos usos indígenas de plantas: “Pelo que vi, os índios, como, em geral, os animais, costumam tomar água não durante a comida, mas depois” ( Baldus, 1950 , p.163). Ao classificar os indígenas junto aos animais, ele circunscreve uma divisão entre animais/indígenas e ocidentais, e questiona se os “narcóticos” (que diz serem “poções embriagantes”), “venerados” pelos “povos naturais”, seriam “degenerados” em “manias” quando utilizados pelos ocidentais. Conclui que são prejudiciais conforme o país e a raça. Dessa maneira, apesar de se interessarem pela planta, exploradores e viajantes, e, por conseguinte, pesquisadores, deturpavam os reais usos, classificações e significados de rituais com a ayahuasca como no Dabucuri, influenciando, ainda, na visão da sociedade ocidental, o que se consolida com a chegada dos salesianos à região do Alto Rio Negro. Entre outras ações relatadas por indígenas, esses missionários empreenderam campanhas de discriminação contra os pajés, quebraram potes com gaapi, confiscaram objetos rituais e destruíram a maloca principal (Diakuru, Kisibi; 2006; Fernandes, 2018 ). Mais recentemente, antropólogos indígenas como a brasileira Rosi Waikon (2016) afirmam que a realização do Dabucuri continua como forma de resistência à criminalização histórica. Assim, a identificação de diversas cosmovisões sobre as plantas é fundamental para discutir, em termos ontológicos, de que ayahuasca falamos hoje, como veremos ( Latour, 2012 ). Esse ponto de partida pode se somar às discussões do renascimento psicodélico, no qual existe mais legitimidade das substâncias psicoativas na ciência e em outras esferas da sociedade, no sentido de qualificar os usos, classificações e significados indígenas da beberagem e das plantas com as quais é preparada.

## Classificações indígenas e a classificação ocidental

Os relatos de exploradores, viajantes e de pesquisas são “camadas” ( Latour, 2020 ) que mostram, por exemplo, que a ayahuasca da qual falavam os exploradores e viajantes era o cipó *Banisteriopsis caapi* , espécie que foi identificada por Spruce no rio Uaupés ( Reichel-Dolmatoff, 1976 ). Distinto, portanto, da ayahuasca que se popularizou nos grandes centros urbanos a partir dos anos 1960, com o advento e expansão das religiões ayahuasqueiras brasileiras. Essas instituições a conhecem, cada qual a seu modo, por nomes como daime (Alto Santo, Santo Daime e Barquinha), hoasca e vegetal (União do Vegetal) (Assis, Rodrigues, 2017; Labate, 2000 ), e nelas são unidos o cipó às folhas da árvore *Psychotria viridis* , planta também amazônica que depois se demonstrou cientificamente possuir propriedades psicoativas potentes quando em sua decocção conjunta ( Strassman, 2019 ).

Entre indígenas, a ayahuasca – que do quéchua, língua franca de parte da floresta amazônica, significa “cipó de morto” (Assis, Rodrigues, 2017) –, do modo como é preparada pelas religiões ayahuasqueiras, está restrita a grupos das montanhas peruanas e equatorianas ( Reichel-Dolmatoff, 1976 ). Diversos povos indígenas da Amazônia, porém, a conhecem por outros nomes, tais como, yagê (siona), caapi (baniwa), kamarampi (ashaninka), kamalãpi (manchineri), nixi pae (kaxinawa) e uni (yawanawa), podendo prepará-la de diversas formas (Assis, Rodrigues, 2017; Tukano, 14 fev. 2019). Dessa maneira, o termo que se popularizou na comunidade científica dos nossos tempos e que utilizamos de modo genérico neste texto não representa a diversidade de espécies de plantas, usos, classificações e significados que indígenas fazem da beberagem ao longo dos tempos.

Se os atores de diferentes épocas estão em um mesmo plano da rede, o exercício para identificar “camadas” da história na qual interagem deve começar do presente em direção ao passado, conforme defende Latour (2020) . Somente assim temos condições de, por meio do renascimento psicodélico, resgatar os relatos de exploradores e viajantes e as pesquisas sobre a ayahuasca no final do século XIX e início do XX, iluminando as transversalidades que perpassam a história longitudinal e entendendo como elas podem, em última instância, ressignificar a ayahuasca e seus usos. Dito pelo autor: “Quando ouvimos segundo a série longitudinal, ela relata uma história maravilhosa; quando a ouvimos segundo a série vertical, ela nos diz ‘como’ temos de compreender toda a história ... para produzir novas” (p.94).

Assim, a partir de nossa referência atual, observamos os estudos do início do século XX, nos quais Reichel-Dolmatoff (1976) e o botânico estadunidense Richard Schultes (1986) afirmam que a utilização de nomes genéricos pelos pesquisadores sobre os usos indígenas de qualquer planta causou certa confusão na literatura especializada, de modo que pode ser difícil saber exatamente de que espécie(s) de *Banisteriopsis* ssp. se estava falando. Como dito, esses estudiosos referenciavam-se nos relatos de exploradores e de viajantes da segunda metade do século XIX. Mesmo assim, os farmacêuticos e químicos brasileiros Oswaldo Costa e Luiz Faria (1936) já indicavam que era possível a existência de outras plantas fornecedoras do yagê.

De todo modo, o que se sabe da descrição e da classificação realizadas pelos exploradores, viajantes e por botânicos leva a pensar que elas foram necessárias para identificar naquele momento que partes do cipó eram utilizadas pelos indígenas e nas suposições dos pesquisadores sobre quais propriedades químicas produziriam os efeitos nos humanos e dos quais podiam ser extraídos os princípios ativos com potenciais terapêuticos. Mostram, ainda, o cenário da chegada da ayahuasca aos grandes centros urbanos via centros de pesquisa nos quais os pesquisadores por um lado demonstravam interesse terapêutico e comercial e, por outro lado, criminalizavam os usos da beberagem por indígenas e mesmo por ocidentais.

A *Revista da Flora Medicinal* foi uma das publicações científicas que abriu espaço para pesquisadores descreverem as propriedades do yagê. Em uma monografia publicada em 1945, o farmacologista brasileiro Jayme Regallo Pereira (1945) utiliza a classificação do farmacologista francês Alexandre Rouhier para indicar o cipó como uma “planta alucinatória”, devido às manifestações na mente. No entanto, a revista era produzida pelo Laboratório da Flora Medicinal, fundado no Rio de Janeiro em 1912 e uma das empresas farmacêuticas brasileiras mais expressivas nos anos 1930 e 1940, que realizava pesquisas com plantas para fins comerciais no mercado nacional e internacional ( Alves, 2005 ), aspecto que aprofundaremos na esteira da discussão sobre o interesse das indústrias farmacêuticas nas substâncias psicoativas hoje.

Costa e Faria (1936) fazem, na mesma revista, ampla descrição de aspectos botânicos e químicos do yagê, incluindo imagens de seus ramos, inflorescência, flor e fruto, mencionando também aspectos históricos e sociais. O texto é uma transcrição do discurso proferido na sede da Associação Brasileira de Farmacêuticos, no Rio de Janeiro, no qual informaram sobre a então chamada *Banisteria caapi* como espécie da família Malpighiaceae, comum em regiões tropicais e subtropicais, particularmente nas Américas. A classificação fora realizada no século XIX pelo botânico alemão August Grisebach, a partir de material coletado por Spruce na região do rio Uaupés (Costa, Faria, 1936).

Isso demonstra o interesse dos latino-americanos pelos estudos sobre as propriedades do cipó, em especial, de brasileiros e de colombianos. Em 1858, o peruano Manoel Villavivencio já havia publicado o livro *Geografia da República do Equador* , em que fazia referências ao caapi. Mas foi o médico colombiano Fischer Cárdenas que isolou, em 1923, um alcaloide e o batizou de “telepatina”, em homenagem ao naturalista colombiano Rafael Zerda Bayon, que havia imaginado sua existência e o relacionado à telepatia. Trabalho semelhante ao que realizou o pesquisador e professor colombiano Antonio Maria Barriga Villalba, que, em 1925, isolou dois alcaloides da planta, dando-lhes outros nomes – yageína e yaginina. Somente dois anos mais tarde, na Europa, o farmacologista francês Louis Lewin também isolou um alcaloide, chamando-o banisterina (Costa, Faria, 1936; Parreiras, 1965 ; Naranjo, 2015 ). A telepatina, a yageína e a banisterina são a mesma harmina que conhecemos hoje ( Reichel-Dolmatoff, 1976 ), mas haveria que verificar se, quimicamente, a yaginina pode ser algum dos outros dois alcaloides presentes no cipó também conhecidos hoje.

A aposta no cipó era tamanha, que no Jardim Botânico do Rio de Janeiro havia exemplares, levados para lá pelo naturalista austríaco Adolpho Ducke, botânico do Museu Paraense Emílio Goeldi. Costa e Faria (1936) informam que a partir dos pequenos caules trazidos por Ducke, cada um com cerca de 2 a 3cm de comprimento por 1 a 1,5cm de diâmetro, na cor pardo-escuro, é que se pode identificar onde se encontra a maior concentração da yageína, e mencionam a possível “aplicação” terapêutica da planta vinda da Amazônia, nomeada como uma “curiosidade científica” no “meio civilizado”.

É preciso considerar que, em 1931, a dimetiltriptamina (DMT) foi sintetizada em laboratório pelo químico canadense Richard Helmut Fredrick Manske a partir de um arbusto norte-americano. Porque o pesquisador deixou o estudo de lado, entretanto, a ligação entre a DMT com as plantas psicoativas ou com o organismo humano só foi identificada nas décadas seguintes. Em 1955, os químicos estadunidenses Fish, Johnson e Horning publicaram o primeiro artigo em língua inglesa em que descreveram a presença da DMT em uma árvore, mas não sabiam que a substância tinha efeito psicoativo. Na mesma década, o químico e psiquiatra húngaro Stephan Szára estudou a respeito da DMT em rapés da Amazônia, sintetizou-a em laboratório e ingeriu algumas doses. No entanto, a DMT continuou como “curiosidade farmacológica” presente em plantas. Apenas em 1965, após já terem sido realizados estudos com camundongos e ratos, é que pesquisadores alemães relataram em artigo na *Nature* ter isolado a DMT no sangue humano, e, em 1972, o bioquímico estadunidense Julius Axelrod informou tê-la encontrado no cérebro humano. Não demorou para que fosse reconhecida como a primeira substância psicoativa endógena humana. Ainda que não fossem tão disseminadas quanto as pesquisas com o ácido lisérgico (LSD), os cientistas investigavam a ação na DMT em distúrbios psicossociais graves, como a psicose, e pensavam que assim estariam mais próximos de um tratamento ( Strassman, 2019 ).

Embora fossem na contramão dos estudos atuais, mais voltados para a DMT, presente nas folhas da árvore *Psychotria viridis* e a qual hoje se sabe ser responsável pelos efeitos na consciência, na percepção e nas emoções quando em composição com o cipó e com seus alcaloides ( Strassman, 2019 ), as pesquisas do início do século XX podem indicar uma necessidade da atenção de pesquisadores da revolução psicodélica com os alcaloides do *Banisteriopsis caapi* . O psiquiatra chileno Claudio Naranjo (2015) defende, a partir de relatos e observações de rituais indígenas, que esses alcaloides também podem conduzir a efeitos psicoativos nos humanos e indica a necessidade de reconhecer a diversidade varietal dos cipós utilizados histórica e atualmente quanto às classificações indígenas e às ocidentais. Ainda, de modo simétrico ao aspecto químico empregado pela ciência ocidental, questionamos o que a cosmovisão indígena diz sobre o que compõe essas plantas; esse pode ser um aspecto mais bem investigado em etnografias.

## A chegada da “planta telepática” à cidade: primeiras pesquisas sobre potenciais terapêuticos e o interesse de eugenistas

Os congressos do renascimento psicodélico reúnem indígenas, religiosos, historiadores, antropólogos, psiquiatras, neurocientistas e demais interessados, por exemplo, nas “curas tradicionais” e em seus potenciais terapêuticos aplicados à medicina ocidental, conforme relata a antropóloga brasileira Beatriz Labate (3 jan. 2017), em análise da programação da Psychedelic Science 2017, conferência realizada, nos EUA, pela Multidisciplinary Association for Psychedelic Studies (Maps) e pela Beckley Foundation. Da mesma maneira que, com frequência, pesquisadores de áreas como a psiquiatria e a neurociência estabelecem associações com instituições e pessoas que fazem uso religioso da ayahuasca na realização de seus estudos e em seus artigos citam usos indígenas e usos religiosos históricos e atuais.

A coexistência dessas temporalidades também é observada nas pesquisas sobre as “aplicações” terapêuticas da ayahuasca no início do século XX, as quais estavam envoltas em controvérsias sobre a origem e os usos descritos pelos exploradores e viajantes do século anterior e o temor de que a planta chegasse à cidade. Em outras palavras, ao se interessar pelos conhecimentos dos indígenas da floresta Amazônica na preparação de “vegetais empregados em medicina” (Costa, Faria, 1936), aqueles pesquisadores também propagavam suas próprias visões de mundo, as quais começavam a constituir a opinião pública de outras esferas da sociedade ocidental daquela época e de hoje.

O trecho citado por Pereira (1945 , p.85) descreve o momento em que se encontram, nas primeiras décadas do século XX, os estudos científicos sobre o yagê.

Há, de um modo geral, no relato dos efeitos provocados pelas plantas alucinatórias uma boa dose de imaginação e fantasia. Quer nos que se entregam aos vícios e às práticas alucinatórias, como nos observadores de tais práticas e até mesmo nos escritores que através de suas publicações divulgam os conhecimentos adquiridos em torno ao problema, em todos, enfim, dificilmente se pode precisar até onde vai a realidade das manifestações psíquicas dos alucinados e onde começa a imaginação criadora dos que relatam os efeitos observados.

Na introdução à sua teoria do ator-rede, Latour (2012 , p.292) afirma que, “na maioria das situações, as ações são afetadas por entidades heterogêneas que não têm a mesma presença local, não se originam na mesma época”. Para compreender, portanto, as temporalidades, as associações e as controvérsias relacionadas à ayahuasca, é necessário colocar em um mesmo plano da rede determinados atores do passado e do presente.

Nesses termos, a ayahuasca pode ser considerada um “híbrido” ( Latour, 1994 , 2012 ), pois congrega elementos da natureza e da cultura, bem como da pré-modernidade e da modernidade, possibilitando que nos questionemos sobre a existência da própria modernidade como uma temporalidade na qual elementos da pré-modernidade não estariam presentes. Com isso, ainda, é possível empreender também um debate político atual, com vistas para as incompreensões do passado e os desafios do futuro, conforme demonstraremos.

Em 1924, em seu artigo “Le yagé: plante télépathique”, Rouhier conta, a partir de relatos, que Bayon dizia que os indígenas ao beber o yagê descreviam com minúcias casas, castelos e cidades que jamais tinham visto presencialmente (Costa, Faria, 1936). Costa e Faria (1936) falam dessas histórias, mas tranquilizam os ocidentais, sugerindo que o yagê não chegaria logo aos centros urbanos, o que não aconteceu, como mostram o advento e a popularização das religiões ayahuasqueiras nas décadas seguintes e até os usos recreativos pela contracultura ( Labate, 2000 ; Strassman, 2019 ).

O Yagê é ingerido pelos curandeiros, pelos adivinhos quando chamados para decidir contendas, descobrir planos do inimigo, aproximação de estrangeiros, apontar quem enfeitiçou um homem doente e para denunciar cônjuges infiéis. Fiquem, porém, tranquilos, meus Senhores, pois o perigo tão cedo não chegará até aqui (Costa, Faria, 1936, p.609).

Na década de 1940, Pereira (1945 , p.101) reconhece que o princípio ativo do cipó, já chamado por ele de harmina, estava “definitivamente incorporado ao arsenal terapêutico” e nota o grande número de publicações, em especial internacionais, sobre o tema. O autor faz um levantamento de alguns dos principais estudos realizados àquela época e os usos científicos em pequenos animais, empreendidos por pesquisadores de laboratórios europeus, também parece entusiasmado com os resultados promissores da harmina no tratamento de tremores patológicos do mal de Parkinson e mesmo com respeito às propriedades telepáticas ( Pereira, 1945 ).

Nessas pesquisas já ficavam evidentes os indícios de que a ayahuasca, naquela época o cipó *Banisteriopsis* spp., pudesse ser útil no tratamento de problemas de saúde pública, que talvez com algum esforço pudéssemos relacionar aos resultados promissores dos usos terapêuticos da ayahuasca no tratamento da ansiedade e da depressão como demonstra hoje uma série de estudos do renascimento psicodélico ( Ona et al., 2019 ; Rocha et al., 2019 ) os quais prometem até revolucionar áreas como a psiquiatria. Afinal, hoje se sabe que os medicamentos alopáticos dessa área médica não apresentam resultados satisfatórios para 1/3 das pessoas que os utilizam, sendo a beberagem e seus compostos químicos candidatos a substituí-los (Santos, Bouso, Hallak, 2020), da mesma maneira que a 3,4-metilenodioximetanfetamina (MDMA), presente no *ecstasy* , vem sendo testada nos EUA no tratamento do estresse pós-traumático entre ex-combatentes de guerra (Beserra, Vieira, 2020).

Ainda sobre as pesquisas com a ayahuasca no início do século XX, chama a atenção o interesse de pesquisadores eugenistas, ligados a ideologias que prosperavam na Europa e no Brasil nas primeiras décadas do século XX e que hoje são condenadas ( Miranda, 2013 ), em particular, na psiquiatria, que naquele momento se institucionalizava em nosso país e, para isso, buscava referências no cenário internacional ( Alarcão, 2018 ; Tarelow, 2018 ). Pereira (1945) faz menção à monografia do psiquiatra brasileiro Ignácio Cunha Lopes, intitulada *A propósito das toxicomanias raras ou menos frequentes entre nós* . Lopes chegou a estudar com Juliano Moreira, um psiquiatra brasileiro bastante conhecido e que tinha algumas ideias controversas ligadas ao eugenismo, e com Lewin, na Alemanha, o que evidencia a associação com pesquisadores europeus no desenvolvimento das primeiras pesquisas sobre a ayahuasca.

Em 1934, Lopes (1934) publica sua monografia em que menciona o caapi na revista *Arquivos Brasileiros de Higiene Mental* , uma das publicações científicas brasileiras responsáveis por difundir os ideais eugenistas, pertencente à controversa Liga Brasileira de Higiene Mental ( Muñoz, 2015 ). Os estudos de Lopes também evidenciam o prestígio com que as ideias eugenistas eram vistas pela rede de pesquisadores da beberagem. Costa e Faria (1936) citam o trabalho dele realizado com o “brilho da sua inteligência” e conclamam a comunidade científica nacional e as autoridades a olhar para os usos terapêuticos do yagê: “Os países de minguados recursos florestais organizam congressos para o estudo das suas plantas. Nós, brasileiros, detentores da maior riqueza vegetal do mundo, ao contrário daqueles, dormimos despreocupadamente” (Costa, Faria, 1936, p.622).

Apesar dos potenciais no tratamento de problemas de saúde pública, os usos terapêuticos por eugenistas alertam sobre os riscos de a ciência utilizar a ayahuasca como forma de controle de corpos e mentes. Àquela época certos manicômios e hospitais europeus, como o Manicômio Nacional da Prússia e o Hospital para Epiléticos, ambos em Postdam, Alemanha, já realizavam pesquisas com a beberagem no tratamento de pacientes, considerados pelos eugenistas, conforme elucida Miranda (2013) , indivíduos inferiores mentalmente. Rudolf Wedel, que apresentou um trabalho sobre o yagê na Sociedade Médico-homeopata Espagírica Alemã, chega a endereçar uma carta ao médico homeopata brasileiro Alcides Nogueira da Silva, recomendando que fossem feitas pesquisas com a beberagem no tratamento da epilepsia e das “doenças nervosas” (Costa, Faria, 1936).

Posto que a ayahuasca poderia ser considerada por psiquiatras eugenistas um novo tratamento para os indivíduos supostamente menos aptos mentalmente, também faz questionar, junto com o historiador brasileiro Vanderlei Sebastião de Souza (2016) , a própria noção de uma eugenia latino-americana mais “suave” em relação à europeia e à estadunidense. O autor argumenta que, embora nos países do hemisfério norte as medidas se baseassem em políticas extremas de segregação racial e controle da reprodução humana, o movimento eugênico brasileiro adotou projetos que repercutiram na sociedade, como as políticas de estímulo ao “branqueamento” da população e a culpabilização dos negros e de outros sujeitos pelo “atraso” civilizacional do país, o que seria resolvido por meio do desenvolvimento científico, como acreditavam.

Conforme mencionado, mesmo com a aposta em suas aplicações clínicas, para os pesquisadores, a ayahuasca demandava cuidado devido à sua origem indígena e aos próprios usos científicos. Costa e Faria (1936 , p.397) a nomeiam entorpecente, que podia ser utilizado com outros fins na cidade:

Mais um entorpecente! Será motivo de alegria ou de tristeza essa revelação? Acreditamos não errar dizendo que, dada a situação subversiva atual do mundo, o nosso sentimento é misto. Alegramo-nos sabendo que a química vai entregar à medicina mais um poderoso agente terapêutico; e entristecemo-nos antevendo a possibilidade da deturpação da sua verdadeira finalidade.

O próprio Lopes (1934 , p.116) entendia que o cipó podia “provocar desordens psíquicas” e teria ações no cérebro: “Diversas plantas da misteriosa Amazônia oferecem e patenteiam ao estudo maravilhas extraordinárias. O iagê figura dentre essas maravilhas que, de tão extraordinárias, podem tornar-se maléficas”. Alerta ainda para a possibilidade de que em breve seus usos científicos, terapêuticos e em contexto urbano poderiam se expandir. O autor faz menção aos usos indígenas para indagar, a partir de citação de trecho da obra *Phantastica* , de Lewin, publicada em 1924, se o cipó estaria associado à “felicidade pessoal”, mas o relaciona aos “selvagens” e à imoralidade do ponto de vista cristão, a nosso ver denotando a partir de qual cosmovisão o uso da beberagem pode ter começado na ciência.

Semelhante à discussão sobre a *Cannabis* ssp., a historiadora brasileira Thamires Moreira (2019) descreve, a partir da leitura do livro de observações do Hospital Nacional dos Alienados, no Rio de Janeiro do início do século XX, que médicos recomendavam os usos terapêuticos da *Cannabis indica* . Mas os usos da planta eram moralizados, pois era associada às populações afro-brasileiras, que a utilizavam com fins medicinais, religiosos e de resistência às imposições morais e legais ocidentais. Por isso, autoridades e pesquisadores estavam mobilizados para enquadrá-la no Código Penal, que previa pena para o charlatanismo. Não muito diferente do que acontece ainda hoje, mesmo na discussão sobre os usos medicinais das propriedades químicas da planta.

Costa e Faria (1936 , p.621) também viram com descrédito o fato de o Laboratório Leysin, de Paris, ter lançado o Cerebroil, em 1929, composto de yageína e indicado para “insuficiência e reeducação cerebral”, depressão nervosa, perda de memória, fobias, “aumento da intensidade do pensamento”: “E nós a pensarmos que só no Brasil é que existe charlatanismo!”. Esse posicionamento evidencia o interesse da indústria farmacêutica na substância psicoativa no início do século XX. Àquela época, a Sandoz já se empenhava por identificar compostos que pudessem ser vendidos à população; foi assim que passou a comercializar o LSD, que Albert Hofmann encontrara em 1938 ( Delmanto, 2020 ; Strassman, 2019 ). Mais recentemente, autores como Beserra e Vieira (2020) chamam a atenção sobre o interesse das indústrias farmacêuticas nas substâncias psicoativas.

No caso da ayahuasca, pontuamos que os usos terapêuticos demandam refletir sobre como isso aconteceria, por exemplo, se por meio do desenvolvimento de microdosagens, como já há pessoas vendendo irregularmente em grupos da internet ( Lima, 2021 ), se estaria acessível no sistema público de saúde e se, para isso, seria submetida como os medicamentos aos trâmites das agências regulatórias, que incluem pesquisas clínicas, precificação etc. Também levam a examinar a quem ela pertence como patrimônio histórico e cultural (Assis, Rodrigues, 2017); se, por exemplo, aos indígenas, então, estes deverão ter direitos sobre parte dos lucros das indústrias.

No entanto, para isso, as indústrias devem entrar na celeuma da criminalização histórica da ayahuasca e de outras substâncias psicoativas, no que diz respeito às “drogas”. O antropólogo brasileiro Júlio Assis Simões (2008) explica que, no senso comum, a categoria “droga” é utilizada para fazer menção às substâncias psicoativas ilícitas, como a maconha, a cocaína e o *ecstasy* , entre outros, cujo uso é visto como um problema de saúde pessoal e coletiva, sendo associado ao crime e à violência e, portanto, deve ser alvo de políticas de controle e proibição. Mas há inconvenientes nessa associação entre drogas com o abuso de psicoativos ilícitos, como a determinação patológica da drogadição e a visão das drogas como uma ameaça à sociedade. Daí o cenário de “guerra às drogas” conforme é colocado pelas políticas proibicionistas, com a propagação da estigmatização, a naturalização da ilegalidade, a potencialização da repressão e a falta de discussão intelectual, ao mesmo tempo que há o favorecimento do crime organizado, do comércio ilegal e do tráfico internacional.

Embora os usos religiosos e os usos científicos da ayahuasca tenham sido regulamentados em 2010 no Brasil, ela não está isenta desse debate. Poucos meses após sua regulamentação, o cartunista Glauco e o filho dele Raoni foram assassinados em uma igreja ayahuasqueira em São Paulo por um frequentador que depois foi diagnosticado com esquizofrenia. O acontecimento mobilizou a opinião pública e fez com que veículos de imprensa, políticos, religiosos e parte da comunidade científica contestassem os usos da beberagem, colocada como uma droga ( Carvalho et al., 2016 ).

Nesse debate, plantas como a coca também sofrem uma criminalização histórica. O psicólogo e antropólogo brasileiro Ivan Farias Barreto (2013) menciona que a folha de coca, utilizada em seu estado natural por povos andinos, é associada pelos ocidentais ao narcotráfico, devido à quantidade de cocaína que, ainda que pouca, detém a planta, sendo objeto de planos políticos que visam coibir sua produção e seu consumo. Quando colocadas sob a mesma categoria indistinta de drogas, a história, os usos, as classificações não ocidentais e a biodiversidade de plantas utilizadas por povos tradicionais são generalizadas, em vez de haver uma discussão séria e livre de preconceitos sobre cada uma. Essas são apenas algumas das camadas pelas quais o presente e o passado se encontram na história e nas quais o renascimento psicodélico pode, de fato, ser um novo momento de reflexão e de discussão sobre as substâncias psicoativas.

## Considerações finais

Os relatos de exploradores e viajantes da segunda metade do século XIX e as pesquisas das primeiras décadas do século seguinte mostram que os autonomeados “civilizados” se interessavam pelas propriedades terapêuticas da ayahuasca no tratamento, por exemplo, de “doenças nervosas”, também por ser uma planta “mágica” que promovia sonhos visionários e visões reveladoras. Um destaque deve ser dado ao interesse de eugenistas na beberagem, utilizada em experimentos com pessoas vistas como um risco à sociedade, bem como ao interesse da indústria farmacêutica no século passado. No entanto, os próprios pesquisadores moralizavam o debate e incitavam a criminalização dos usos indígenas e na cidade, pois a planta poderia “viciar” os ocidentais, inclusive, como uma “vingança” mandada pelos povos explorados.

O aspecto histórico põe luz sobre o debate político atual a respeito da ayahuasca, no que diz respeito a diversas questões abordadas neste artigo, como o enfrentamento de um quadro epistemológico que moraliza e criminaliza os usos indígenas e coloniza as classificações, reduzindo as espécies de plantas, propriedades químicas e os significados aos parâmetros ocidentais; a problematização do renascimento psicodélico, em especial, sobre os potenciais terapêuticos das substâncias psicoativas como um possível tratamento para problemas de saúde pública e o cuidado para não haver a busca do controle de corpos e mentes; o interesse da indústria farmacêutica na beberagem e em suas propriedades terapêuticas e a necessidade de regulação desses usos; e um debate sobre as drogas que discuta o próprio proibicionismo e seus efeitos na sociedade e inclua no diálogo pesquisadores que há anos realizam estudos e avaliações de políticas públicas nesse campo.

Ainda, pesquisas etnográficas com diferentes agrupamentos indígenas podem informar sobre conhecimentos da diversidade varietal de plantas utilizadas no preparo da ayahuasca e, na simetria com a química ocidental, sobre o que compõe essas plantas na cosmovisão indígena. Todos esses pontos destacados podem ser eixos norteadores para uma agenda de pesquisa sobre a ayahuasca e outras substâncias psicoativas, particularmente aquelas utilizadas por populações tradicionais.

Nesse sentido, uma leitura do aspecto histórico da teoria do ator-rede parece ser programática, ao trazer elementos teórico-metodológicos para pensar a rede e os atores, associações, controvérsias, camadas e temporalidades que a compõem, incluindo nessa rede pesquisadores da história das ciências e da história indígena. Foi dessa maneira que identificamos que as contradições sobre a ayahuasca não são tão recentes e encontramos elementos pelos quais propomos construir, alicerçados nas experiências do ontem, uma nova história, não apenas para a ciência ocidental que se interessa por seus potenciais terapêuticos, mas para os próprios indígenas que usam a beberagem há milênios. Afinal, embora atualmente a ayahuasca se mostre mais legitimada no debate público, conhecer seu passado pode mostrar que, ainda que as substâncias psicoativas sejam vistas como um possível agente de uma revolução em áreas como a psiquiatria, há que enfrentar na esfera política incompreensões que não são de agora.

## NOTAS

1 Utilizamos o termo ayahuasca, que é o mais difundido pela ciência na atualidade. No entanto, ao longo do texto discutimos essa categorização ocidental.

2 Optamos por utilizar a grafia das palavras e as construções das frases como encontradas nos documentos.

3 Reichel-Dolmadoff (1976) menciona 1851, mas Costa e Faria (1936) dizem que foi em 1853; há ainda outras datas citadas para esse acontecimento ( Schultes, 1986 ).
