# Supplementary material for: Relatos de exploradores e viajantes e primeiras pesquisas científicas com a ayahuasca, 1850-1950, no debate atual sobre o “renascimento psicodélico”
Source: Hist Cienc Saude Manguinhos. 2023 Jul 10;30:e2023023. [Article in Portuguese] doi: 10.1590/S0104-59702023000100023 (PMC10395591; doi:10.1590/S0104-59702023000100023)
Supplement: Translation

# Translation: Reports by explorers and travelers and the first scientific studies on ayahuasca (dating from 1850 to 1950) within the current debate on the “psychedelic renaissance”

**DOI:** 10.1590/S0104-59702023000100023en

**Authors:** Vinícius Maurício de Lima, Maria Gabriela Silva Martins da Cunha Marinho

**Affiliations:** i Brazil Doctoral candidate, Graduate Program in Human and Social Sciences/Universidade Federal do ABC. São Bernardo do Campo – SP – Brazil vmlima9@gmail.com; ii Brazil Professor and researcher, Graduate Program in Human and Social Sciences/Universidade Federal do ABC; associate researcher, School of Medicine History Museum/Universidade de São Paulo. São Bernardo do Campo – SP – Brazil gabriela.marinho@ufabc.edu.br

**Keywords:** ayahuasca, history of science, indigenous history, psychedelic renaissance, drugs

## Abstract

This article describes the associations and controversies between indigenous and western uses of ayahuasca between 1850 and 1950 in relation to the “psychedelic renaissance.” This movement has gained scientific attention since 2000, but hearkens back to the 1960s and 1970s, when anti-drug policy halted research on the “therapeutic potential” of psychoactive substances. Pioneering studies on ayahuasca date back to the early twentieth century and mention reports of expeditions to Amazonia from 1850 onward. Here, these articles and reports are analyzed according to the historical aspect of actor-network theory and recent studies. We infer that history casts light on the current political debate about indigenous uses, classifications, and meanings, pharmaceutical interest in ayahuasca, and the debate on “drugs.”

Ayahuasca^1^ is a beverage that has been used for thousands of years by indigenous people in the Amazonian highlands and lowlands (currently Bolivia, Brazil, Colombia, Ecuador, Peru, and Venezuela). It is estimated that over one hundred indigenous nations currently use this herbal substance in their rituals (Assis, Rodrigues, 2017; Tukano, 14 fev. 2019). From the second half of the twentieth century it has become popular in Brazil and abroad with the advent and expansion of Brazilian religions involving ayahuasca and neo-ayahuasca groups, which differ from the religious groups by being “universalist” or attempting to disconnect themselves from religious precepts ( Labate, 2000 ; Lima, 2021 ). As a result of the anti-drug policies of the 1960s and 1970s, the institutions and people who utilized ayahuasca were persecuted for decades by government agents, other religious institutions, and the media ( Antunes, 2012 ). Research on the “therapeutic potential” of ayahuasca and its chemical compounds (as well as other psychoactive substances) was interrupted, and researchers only began to return to the subject in the 1990s ( Grob et al., 2012 ; Strassman, 2019 ).

But it was the regulation of religious and scientific uses of ayahuasca in countries like the United States and Brazil during the 2000s and 2010s that recognized indigenous and religious uses of this substance, marking the “renaissance” of “psychedelic science” in areas like psychiatry and neuroscience. This is especially due to growth in investment, in the number of clinical trials, and in international events on the therapeutic uses of ayahuasca and other “psychedelics” ( Antunes, 2012 ; Beserra, Vieira, 2020; Guimarães, 20 jul. 2017; Leite, 21 dez. 2020; Rodrigues, Beserra, 2020). The term “renaissance” refers back to the studies conducted in the 1960s and 1970s that were suspended as a result of prohibitionist policies.

This mention of the 1960s and 1970s led us to question whether the first studies on the therapeutic potential of ayahuasca date back to this period or even earlier. This article demonstrates that even though the term “psychedelic” was coined by the English psychiatrist Humphry Osmond (in letters he exchanged with his countryman, the writer Aldous Huxley) during the 1950s and proliferated during the 1960s and 1970s in science and in the counterculture movement, showing the relevance of the topic at that time ( Delmanto, 2020 ), research was conducted in the 1930s and 1940s on the therapeutic potential of ayahuasca (known at the time as *yagê* or *caapi* ). To conduct their studies at that time, researchers looked for information in reports from explorers and travelers who participated in expeditions to the Amazon region during the second half of the nineteenth century.

Indigenous rituals like the Dabucuri, which uses *caapi* (or *gaapi* , as we shall see), were mentioned in several reports, such as one by the British naturalist Alfred Russel Wallace, who like Charles Darwin also theorized that evolution took place through natural selection. This information served as a reference for research by botanists, pharmacists, and even eugenicist psychiatrists interested in its “applications.” Despite their controversial interests, these studies indicated that ayahuasca could be useful in treating public health problems such as anxiety and depression, as the current literature shows ( Ona et al., 2019 ; Rocha et al., 2019 ).

Still, considering the reflections by the Brazilian philosopher Pedro Paulo Pimenta (2020) on the relationship between humans and plants in the west, the early studies removed the indigenous uses, classifications, and meanings of ayahuasca, reducing them to an epistemological framework based on the natural sciences and a single concept of humanity (the modern one) via diffusion of therapeutic uses. Not even the ethnographic studies conducted during the first half of the twentieth century detailed indigenous cultural aspects, as observed by the Austro-Columbian anthropologist Gerardo Reichel-Dolmatoff (1976) . After all, they did not address the meanings of the plants that comprise ayahuasca or even ayahuasca itself, as most recent studies by indigenous researchers that have positioned them as fundamental in the constitution of indigenous subjects, for example, have shown (Diakuru, Kisibi, 2006; Fernandes, 2018 ).

The first census of “drug addicts” conducted throughout Brazil in 1962 stated that ayahuasca did not present “a significant sociomedical problem”^2^ like other psychoactive substances such as *Cannabis* spp. ( Parreiras, 1965 , p.44). But reports from explorers and travelers and studies from the early twentieth century reveal, in the words of the French philosopher, sociologist, and anthropologist Bruno Latour (2012) , a diversified “network” of associations and scientific and social controversies about the indigenous and western uses of the “telepathic plant” that reached Rio de Janeiro, the capital of Brazil at that time. Researchers posed moral and legal questions about this beverage of indigenous origin that could “pervert” westerners, propagating prejudices about the uses of ayahuasca by indigenous people and westerners that endure until today.

In this sense, our goal in this article is to describe the associations and controversies between indigenous and western uses of ayahuasca between 1850 and 1950 and how they relate to the psychedelic renaissance. To do so, we investigate reports by explorers and travelers in the second half of the nineteenth century, particularly a report first published by Wallace (2004) in 1889 in *Viagens pelo Amazonas e rio Negro* (Travels in Amazonas and the Rio Negro) and four scientific articles published in three Brazilian scientific journals during the first half of the nineteenth century which were provided by the Brazilian Center for Information on Psychotropic Drugs at the University of São Paulo; we also include contemporary literature for discussion.

From these reports and articles we propose analyzing the historical aspect of Latour’s actor-network theory (2012), which is more widely used in the social sciences but (as we shall demonstrate) can also be applied in history and historiography. Our approach considers three topics of this theory for analysis. From the first topic, we consider that indigenous people, explorers, travelers, and researchers in the late nineteenth and early twentieth centuries were located within the same “network” (in Latourian terms) as modern indigenous people, researchers, and other actors, and established associations and controversies that show us the uses, classifications, and meanings of ayahuasca over the years.

The second topic allows us to observe the development of some “layers” ( Latour, 2020 ), events through which we reveal that previous researchers only related ayahuasca to the vining plant *Banisteriopsis caapi* . This differs from the ayahuasca described by religious groups, which combines this vine with the leaves of *Psychotria viridis* , a tree that is the object of most current studies on the chemical potential of ayahuasca. These events include reports by travelers and explorers on indigenous uses, as well as reports by modern indigenous anthropologists and studies from the early twentieth century and the modern era, and may indicate the need for new research on the therapeutic potential of this vine.

Finally, the third topic allows us to identify different temporalities to demonstrate that pre-modern and modern elements coexist in the past and in the present, since ayahuasca brings them together as an actor within the network itself, or as a “hybrid” according to Latour (1994) , and offers elements for the political discussion ( Latour, 2012 ) necessary for the psychedelic renaissance to take place. These elements include the criminalization of indigenous rituals such as the Dabucuri; addressing an epistemological landscape that removes indigenous uses, classifications, and meanings; pharmaceutical companies’ interest in ayahuasca and its chemical properties; the therapeutic uses of ayahuasca in controlling bodies and minds; and the debate on “drugs.”

## Reports of ayahuasca use and the criminalization of indigenous rituals

For centuries, explorers, travelers, and researchers (especially from Europe) in areas like botany, chemistry, pharmacology and ethnology were interested in how indigenous people in South America used certain plants, placing all these actors into a single network in which they established relatively stable links indicating the motives that joined them or contradictions that distanced them from each other ( Latour, 2012 ). In his text “The use of wild plants in Tropical South America,” the Belgian anthropologist Claude Lévi-Strauss (1986 , p.44) collected a variety of knowledge about some of the plants used by indigenous peoples in this region, for example to make ointments and medicines, and stated that “few primitive people have acquired as complete a knowledge of their botanical environment as the South American Indian.”

There was little scientific interest in the properties of plants with psychoactive components, however; this began to change especially in the 1890s after the discovery of mescaline, the chemical compound in the peyote cactus found in the Americas. But scientific interest in mescaline was restricted, and few articles were published prior to the 1930s ( Strassman, 2019 ).

As for ayahuasca, the first missionary reports of indigenous use of this herbal brew in the Amazon region appeared in texts by two Jesuits in the late seventeenth and early eighteenth centuries. The Spaniard José Chantre y Herrera spoke of a “diabolical beverage,” and the Swiss missionary Juan Magnin said he had learned of an indigenous medicine. Also in the eighteenth century, the Jesuit Pablo Maroni described an “intoxicating drink” and the Jesuit Franz Xaver Veigl described its use. Meanwhile the first traveler reports date back to the early nineteenth century, when the Swiss explorer and naturalist Johann Jakob von Tschudi encountered indigenous people in Peru who used ayahuasca ( Schultes, 1986 ). Although their comments were brief, reports by these and other explorers and travelers were popular in the academic literature about the therapeutic potential of ayahuasca in the early twentieth century.

Among the reports from nineteenth-century travelers, Antunes (2011) highlights those by the British physician and naturalist Richard Spruce as “a paradigmatic case” because he was widely cited in subsequent works. Spruce spent 15 years traveling in the Amazon in the nineteenth century; he spent some time among peoples including the Guajibos, Záparos, and Tukanos and is thought to be the first to harvest *caapi* (in the early 1850s)^3^ and send it to European herbariums for study. His observations were only reported in 1873 and reached the public in 1908 with the publication of his two-volume book *Notes of a botanist on the Amazon and Andes* , edited by Wallace (Costa, Faria, 1936; Schultes, 1986 ).

But these reports were not always ethnographically substantial, and often misrepresented the indigenous uses, classifications, and meanings of ayahuasca. For example, they stated that indigenous people like the Tarianas, Záparos, and Shuaras (generally known as Jíbaros to westerners) used “hallucinogens” or “poisons” with “toxic” effects as “remedies,” which also allowed them to dream and practice telepathy and clairvoyance. Spruce, for instance, recalled his time in the Alto Rio Negro and mentioned the Dabucuri ritual performed by various indigenous nations that lived and still live in northwestern Amazonia along the banks of the Uaupés River, but did not offer profound analysis (Costa, Faria, 1936).

The Dabucuri (from the Nheengatu language spoken throughout the Rio Negro region) is a ritual stretching back thousands of years among the indigenous people of the Alto Rio Negro that combines knowledge such as narratives of human creation, ancestry, and the origins of the rural areas. It also involves rites of passage, political alliances, and marriage arrangements. Between dances, songs, and the sounds of musical instruments like flutes, a community from the same clan or nation offers a large quantity of seasonal fruit, fish, or objects (baskets or the basket-like press known as a *tipiti* ) and is received by another group with distribution of *caxiri* (a fermented drink made from cassava and consumed in large quantities during the ritual), *caapi* , and tobacco; afterward, the recipient group is expected to reciprocate. Today 23 ethnic groups from four language families (Arawak, Maku, Tukano, and Yanomami) practice this ritual regularly, each in their own way (Diakuru, Kisibi, 2006; Waikon, 2016 ).

Reports of this ceremony were incorporated by researchers, and were also a way of expressing ethnocentrism. Although the reports by Spruce (who traveled in Amazonia with Wallace) are relevant, Wallace’s are also interesting; in his 1889 book *Viagens pelo Amazonas e rio Negro* , Wallace described his first visit to the Uaupés River with a white interlocutor (not Spruce in this report) and three indigenous people. He brought western objects with him to trade with the indigenous people for plants, animals, and other objects, and described his delight in meeting “the true representatives of the forest,” people of “semi-civilized races” (Wallace, 2004).

At Wallace’s request, a native named Calixto, the leader of one of the groups he met, determined that the ritual would be held and that the indigenous people would drink *caxiri* and *caapi* . Wallace described approximately three hundred people (adult men and women, young people of both genders, and children) who participated, painting and adorning their bodies with feathers and preparing for the ritual. The women made the *caxiri* with water they collected from the river and the liquid from cassava harvested in the planted areas around the huts, and collected dry branches from the forest to light the fires. Meanwhile, the men wove crowns from vegetation and made other objects.

During the ceremony, which began at sundown, young men and women danced to the sound of drums and flutes and drank *caxiri* in the central longhouse. At this point what Wallace called the “snake dance” began, part of an initiation ritual for the young men in which each carried two enormous snakes made of *imbaúba* wood. They danced at the door of the longhouse with the snakes on their shoulders, imitating the animal’s movements, until finally entering. Once inside the longhouse the snakes fought each other, while other people distributed the *caxiri* to those who were present. Next, the young men set aside the snakes and began to distribute the *caxiri* .

Later the *caapi* was introduced, distributed by an older indigenous man in the middle of the longhouse, from a large clay vessel using two small gourds. Young men with bows, arrows, and spears came in pairs to receive the “excessively bitter” brew. Despite making it clear that he drank and liked the *caxiri* , Wallace did not confirm whether he drank the *caapi.* Furthermore, although he expressed interest in many of the plants he encountered during his narrative, Wallace did not make any effort to understand the relationships these peoples established with each one, nor did he describe the species of plants utilized or the preparation or meaning of the uses of *caapi* .

The network, however, is not restricted to the researchers and indigenous people of the past, and encounters a generation of modern indigenous researchers, important actors in the modern field of study on ayahuasca ( Latour, 2012 ) who conduct the current discussion on “localized knowledge,” in which the variety of positions is an alternative to relativism and indicates that knowledge is always partial and localizable. In other words, by being defined situationally, these connections allow us to consider “networks of differential positions” in which the self is resignified when it resembles the other: in this case, in studies conducted by individuals with distinct cosmovisions ( Haraway, 1995 ).

In this way, the Desana people (also from the Uaupés River) explain that the Dabucuri is a ritual that involves the entire community, where indigenous visitors are received to celebrate the land, the rivers, the trees, the animals, and other beings at certain times of the year, along with their interconnections with their relatives and/or in-laws, their ancestors, with other peoples (fish-people, plant-people etc.), and especially to celebrate the advent of humanity, which they explain as having emerged through the creation myth involving the divinity Gaapi. The indigenous people descend from Gaapi, and are connected to him through the plant *gaapi* , a metonym for the divinity. This knowledge is carefully transmitted to the new generations, which continue the “ *gaapi* -people” (Diakuru, Kisibi, 2006).

In his autoethnographic description, the indigenous anthropologist Jaime Moura Fernandes (2018) explains that among the Desana, there are three types of *gaapi* : the child-of-the-day *gaapi* used in rites of passage for young people of both sexes, the fruit *gaapi* consumed during the Dabucuri rituals, and the *Waimahsã gaapi* consumed by the *kumuã* (healers) during the fish Dabucuri; the latter two types of *gaapi* are not cultivated by humans, but rather exist “spontaneously” in the forest. He also mentions the extinct *gaapi* that before the large floods were used by specialists to “take action for life” and to foresee dangers and prevent disease. But he does not say whether each *gaapi* is produced from a unique morphophysiological species, just that *gaapi* is prepared with *patʉ* or *ipadu* ( *Erythroxylum novogranatense* ), a tree in family Erythroxylaceae with leaves similar to coca: “When *gaapi* is not mixed it does not produce the desired effect” ( Fernandes, 2018 , p.57).

*Gaapi* is more than a hallucinogenic plant; it is the divinity from whom all the Desana are descended. So while Wallace (2004) linked *caapi* to animosity among the indigenous people who drank it and became “enraged,” running “furiously” around the longhouse as if they were about to kill an enemy and denoting “savagery,” Fernandes (2018) posits that the indigenous people react ritually with “violence” when they drink the beverage because during the event the *gaapi* connects the *gaapi* -people of the past and the present.

From the former viewpoint, plants are presented as a secondary kingdom, in line with how western natural and biological history classifies them in relation to the animal kingdom and ultimately to *Homo sapiens* ( Pimenta, 2020 ), which uses these plants as “narcotics.” Meanwhile, the latter viewpoint considers plants to be decisive in the lives of humans, like the *timbó* is to the Suruwahas, whose ways of life and death are intertwined with this toxic leguminous plant ( Aparicio, 2019 ).

In the studies from the first half of the twentieth century, even an ethnologist could not overcome the western perspective on ayahuasca; the German-Brazilian Herbert Baldus wrote a 1950 article entitled “Bebidas e narcóticos dos índios do Brasil: sugestões para pesquisas etnográficas” (Drinks and narcotics of the Indians of Brazil: suggestions for ethnographic studies) for the journal *Sociologia* , in which he stated the following on indigenous use of plants: “From what I have seen, the Indians, like the animals in general, tend to drink water not while they eat, but after” ( Baldus, 1950 , p.163). In classifying indigenous peoples alongside animals, Baldus drew a line between animals/indigenous people and westerners, and questions whether “narcotics” (which he describes as “intoxicating potions”) “revered” by “natural people” would “degenerate” into “manias” when utilized by westerners. He concluded they were harmful depending on country and race. In this way, although explorers, travelers, and later researchers were interested in the plant, they misrepresented the real uses, classifications, and meanings of ayahuasca rituals like the Dabucuri, and also influenced the view of western society, which solidified when the Salesians arrived in the Alto Rio Negro. Among other acts reported by the indigenous people, these missionaries carried out discriminatory campaigns against leaders, smashed vessels containing *gaapi* , confiscated ritual objects, and destroyed longhouses (Diakuru, Kisibi; 2006; Fernandes, 2018 ). More recently, indigenous anthropologists like the Brazilian Rosi Waikon (2016) have stated that the Dabucuri ritual is still performed as a way of resisting the criminalization of the past. Identifying the various cosmovisions involving plants is consequently essential to debate (in ontological terms) what ayahuasca we are talking about today, as we shall see ( Latour, 2012 ). This starting point can be combined with discussions on the psychedelic renaissance, where psychoactive substances have taken on greater legitimacy in science and in other spheres of society, in terms of qualifying the indigenous uses, classifications, and meanings of this brew and of the plants with which it is prepared.

## Indigenous classifications and western classification

The reports by explorers, travelers, and studies are “layers” ( Latour, 2020 ) which show that the ayahuasca described by explorers and travelers was the vine *Banisteriopsis caapi* , for example, a species identified by Spruce along the Uaupés River ( Reichel-Dolmatoff, 1976 ). This differed from the ayahuasca that became popular in the large cities in the 1960s, with the advent and expansion of Brazilian ayahuasca religions. These institutions each know it in their own way by names like *daime* (in the Alto Santo, Santo Daime, and Barquinha religions), *hoasca* , and *vegetal* (União do Vegetal) (Assis, Rodrigues, 2017; Labate, 2000 ); they combine the vine with the leaves of *Psychotria viridis* , an Amazonian tree that was later scientifically shown to have potent psychoactive properties in this combined brew ( Strassman, 2019 ).

Use of ayahuasca (from Quechua, the lingua franca of part of the Amazon Forest, meaning “vine of the dead”) (Assis, Rodrigues, 2017) among indigenous people in the form it is prepared by ayahuasca religions is limited to groups in the Peruvian and Ecuadorian mountains ( Reichel-Dolmatoff, 1976 ). But various indigenous peoples in the Amazon know it by other names like *yagê* (Siona), *caapi* (Baniwa), *kamarampi* (Ashaninka), *kamalãp* i (Manchineri), *nixi pae* (Kaxinawa), and *uni* (Yawanawa), and prepare it in various ways (Assis, Rodrigues, 2017; Tukano, 14 fev. 2019). As a result, the term that became popular among the modern scientific community and is used generically in this text does not represent the diversity of plant species, uses, classifications, and meanings that indigenous people have attached to this beverage over time.

If actors from different eras are located within the same network, our work to identify “layers” of history in which they interact should begin in the present and look backward, according to Latour (2020) . Only in this way will we be able to reexamine (through the psychedelic renaissance) the reports of explorers and travelers and studies on ayahuasca from the late nineteenth and early twentieth centuries, casting light on the transversalities that extend beyond longitudinal history and understanding how they can ultimately give new meaning to ayahuasca and its uses. As Latour (2020 , p.94) states: “Understood according to the longitudinal series, it tells a wondrous story; listened to according to vertical series, it tells us how we have to understand any story of salvation — so that we can produce new ones.”

So from our current reference, we observe the studies from the early twentieth century in which Reichel-Dolmatoff (1976) and the American botanist Richard Schultes (1986) stated that the use of genus names by researchers addressing the indigenous uses of any plant caused a certain degree of confusion in the specialized literature, to the extent that it may be difficult to determine exactly what species of *Banisteriopsis* spp. are being referred to. As mentioned, these scholars were referring to the reports by explorers and travelers from the latter half of the nineteenth century. Still, the Brazilian pharmacists and chemists Oswaldo Costa and Luiz Faria (1936) had already indicated the potential existence of other plants that produced *yagê* .

In any case, what is known about the descriptions and classifications by travelers, explorers, and botanists leads us to consider whether they were necessary at that time to identify which parts of the vine were used by the indigenous people and in the researchers’ assumptions on which chemical properties produced its effects on humans and contained active ingredients with therapeutic potential that could be extracted. They also showed the scenario in which ayahuasca reached the large cities via research centers, in which the researchers simultaneously demonstrated therapeutic and commercial interest while also criminalizing its use by indigenous people and even by westerners.

The *Revista da Flora Medicinal* was one of the scientific publications in which researchers described the properties of *yagê.* In a 1945 monograph, the Brazilian pharmacologist Jayme Regallo Pereira (1945) used the classification by the French pharmacologist Alexandre Rouhier to indicate the vine was a “hallucinatory plant” due to its mental manifestations. However, the journal was produced by the Medicinal Flora Laboratory, founded in Rio de Janeiro in 1912 and one of the country’s most significant pharmaceutical companies during the 1930s and 1940s; it conducted research on plants for commercial uses in the domestic and international markets ( Alves, 2005 ), an aspect we shall return to in more detail later in the discussion on current interest in psychoactive substances among pharmaceutical manufacturers.

In the same journal, Costa and Faria (1936) offer an ample description of the botanical and chemical aspects of *yagê* , including images of its branches, inflorescences, flowers, and fruit, and also touch on historical and social aspects. The text is a transcript of a speech given at the headquarters of the Brazilian Pharmaceutical Association in Rio de Janeiro, in which they reported on what at that time was called *Banisteria caapi* as a species in family Malpighiaceae, common in tropical and subtropical regions, particularly in the Americas. This classification was made in the nineteenth century by the German botanist August Grisebach using material collected by Spruce in the region of the Uaupés River (Costa, Faria, 1936).

This demonstrates Latin American interest in studies on the properties of the vine, particularly among Brazilians and Colombians. In 1858, the Peruvian Manoel Villavivencio had already published a book entitled *Geografia da República do Equador* (The geography of the Republic of Ecuador) which mentioned *caapi* . But it was the Colombian physician Fischer Cárdenas who isolated an alkaloid in 1923 and dubbed it “telepatina” in an homage to the Colombian naturalist Rafael Zerda Bayon, who had imagined its existence and linked it to telepathy. Similar work was done by the Colombian research and professor Antonio Maria Barriga Villalba, who isolated two alkaloids from the plant in 1925, and gave them other names: yageina and yaginina. Only two years later in Europe, the French pharmacologist Louis Lewin also isolated an alkaloid and called it banisterina (Costa, Faria, 1936; Parreiras, 1965 ; Naranjo, 2015 ). Telepatina, yageina, and banisterina are all the same harmine that is currently known ( Reichel-Dolmatoff, 1976 ), but it was necessary to chemically determine whether yaginina could be one of the other two alkaloids present in the vine which we also know today.

Interest in the vine was such that the Rio de Janeiro Botanical Garden contained specimens brought there by the Austrian naturalist Adolpho Ducke, a botanist at the Museu Paraense Emílio Goeldi. Costa and Faria (1936) reported that they were able to use these small, dark brown stalks Ducke brought (each roughly 2 to 3cm long and 1 to 1.5cm in diameter) to identify where the greatest concentration of yageina was found, and mentioned potentially therapeutic “application” of this Amazonian plant which they called a “scientific curiosity” in the “civilized environment.”

We should note that in 1931 dimethyltryptamine (DMT) was synthesized in the laboratory from a North American shrub by the Canadian chemist Richard Helmut Fredrick Manske. But because this researcher did not continue with his study, the link between DMT and psychoactive plants or human beings was only identified in the following decades. In 1955, the American chemists Fish, Johnson and Horning published the first book in English describing the presence of DMT in a tree, but they did not know that this substance had psychoactive effects. In that same decade, the Hungarian chemist and psychiatrist Stephan Szára studied DMT in snuff from Amazonia, synthesized it in the laboratory, and took several doses. However, DMT remained a “pharmacological curiosity” present in plants. Only in 1965, after mouse and rat studies, German researchers reported isolating DMT in human blood in an article in *Nature,* and in 1972 the American biochemist Julius Axelrod reported finding it in the human brain. It was not long before it was recognized as the first endogenous psychoactive substance in humans. Although they were not as widely published as studies on lysergic acid diethylamide (LSD), scientists investigated the action of DMT in severe psychosocial disorders such as psychosis, and believed they were close to finding a treatment ( Strassman, 2019 ).

Although they ran counter to current studies that focus more on the DMT present in the leaves of the tree *Psychotria viridis,* which today we know is responsible for its effects on consciousness, perception, and emotions in compounds with ayahuasca root and its alkaloids ( Strassman, 2019 ), studies from the early twentieth century may indicate that researchers involved in the psychedelic revolution need to be attentive to the alkaloids in *Banisteriopsis caapi* . The Chilean psychiatrist Claudio Naranjo (2015) states from reports and observations of indigenous rituals that these alkaloids can also cause psychoactive effects in humans, and indicates the need to recognize the diversity of the vines used in the past and today, in terms of indigenous as well as western classifications. Furthermore, alongside the chemical aspect employed by western science, we question what the indigenous cosmovision says about the makeup of these plants: this could be addressed further in ethnographies.

## The “telepathic plant” arrives in the city: early studies on therapeutic potential and interest among eugenicists

Congresses on the psychedelic renaissance bring together indigenous people, religious practitioners, historians, anthropologists, psychiatrists, neuroscientists, and others interested in “traditional cures” and their therapeutic potential when applied to western medicine, as described by the Brazilian anthropologist Beatriz Labate (3 jan. 2017) in an analysis of the program of events for Psychedelic Science 2017, an event held in the United States by the Multidisciplinary Association for Psychedelic Studies (MAPS) and the Beckley Foundation. This is similar to how researchers in areas like psychiatry and neuroscience often establish associations with institutions and people who use ayahuasca for religious purposes to conduct their studies, and cite indigenous uses and past and present religious uses in their articles.

The coexistence of these temporalities can also be seen in studies on the therapeutic “applications” of ayahuasca at the start of the twentieth century, which were surrounded by controversy about the origin and the uses described by the explorers and travelers from the previous century and fears that the plant would reach the cities. In other words, by taking interest in the knowledge of indigenous people from the Amazon Forest in preparing the “plants used in medicine” (Costa, Faria, 1936), these researchers also propagated their own worldviews, which began to shape public opinion in other spheres of western society, then as well as today.

The passage cited by Pereira (1945 , p.85) describes the first scientific studies on *yagê* from the early decades of the twentieth century:

In general, there is a large portion of imagination and fantasy in reports of the effects produced by hallucinatory plants. Whether in those that give themselves over to vices and hallucinatory practices, in us as the observers of such practices and even we writers who publish the knowledge acquired about the problem in our publications, ultimately in all cases it is difficult to specify how far the reality of the psychic manifestations of the hallucinators extends, and where the creative imagination of those who report these observed effects begins.

In the introduction to his actor-network theory, Latour (2012 , p.292) states that “In most situations, actions are affected by heterogeneous entities that do not have the same local presence and do not originate from the same era.” So to understand the temporalities, associations, and controversies related to ayahuasca, we must place certain actors from the past and the present onto the same plane of the network.

In these terms, ayahuasca can be considered a “hybrid” ( Latour, 1994 , 2012 ), since it combines elements of nature and culture, as well as of pre-modernity and modernity, allowing us to question the existence of modernity itself if elements of pre-modernity are not present. Consequently, a current political debate can also be employed with regard to the misunderstandings of the past and the challenges of the future, as we shall see.

In a 1924 article entitled “Le yagé: plante télépathique” (Yagê: telepathic plant), Rouhier cited reports by Bayon stating that indigenous people who drank *yagê* described houses, castles, and cities they had never seen in person in minute detail (Costa, Faria, 1936). Costa and Faria (1936) mentioned these stories but reassure western readers by suggesting that *yagê* would not reach urban centers anytime soon, which was not the case, with the advent of ayahuasca religions in the following decades and even recreational use in the counterculture ( Labate, 2000 ; Strassman, 2019 ).

*Yagê* is ingested by the healers, by the seers when they are called to resolve disputes, discover the plans of the enemy, the arrival of outsiders, to indicate who bewitched a sick man, and to report unfaithful spouses. But stay calm, Gentlemen, since the danger will not arrive here anytime soon (Costa, Faria, 1936, p.609).

In the 1940s, Pereira (1945 , p.101) recognized that the main active ingredient in the root, which he already called harmine, was “definitively incorporated into the therapeutic arsenal” and noted the large number of publications (especially international ones) on this topic. The author surveyed some of the main studies of that time and scientific use in small animals by researchers in European labs, and also seemed enthusiastic about the promising results of harmine in treating pathological tremors from Parkinson’s disease and even with regard to its telepathic properties.

These studies already contained clear signs that ayahuasca (at that time, the vine *Banisteriopsis* spp.) could be useful in treating public health problems, which perhaps with some effort could be linked to the promising therapeutic results of ayahuasca in treating anxiety and depression, as a series of studies in the current psychedelic renaissance have shown ( Ona et al., 2019 ; Rocha et al., 2019 ), and even promise to revolutionize areas such as psychiatry. After all, today we know that allopathic medications in this field of medicine do not yield satisfactory results for a third of patients who use them, and this herbal compound and its chemical components are candidates to replace them (Santos, Bouso, Hallak, 2020), just as the 3,4-methylenodioxy-methamphetamine (MDMA) in ecstasy has been tested in the US to treat post-traumatic stress syndrome in combat veterans (Beserra, Vieira, 2020).

Also notable about research on ayahuasca in the early twentieth century is interest among eugenicist researchers linked to ideologies that flourished in Europe and Brazil at that time but today are considered reprehensible ( Miranda, 2013 ), particularly in the field of psychiatry, which was becoming institutionalized in Brazil at that time and looked to references from abroad ( Alarcão, 2018 ; Tarelow, 2018 ). Pereira (1945) mentioned a monograph by the Brazilian psychiatrist Ignácio Cunha Lopes entitled “A propósito das toxicomanias raras ou menos frequentes entre nós” (On rare or less common addictions among us). Lopes studied with Juliano Moreira, a well-known Brazilian psychiatrist who held some controversial ideas related to eugenics; like Lewin he also studied in Germany, showing the connection between European researchers and the first studies on ayahuasca.

In 1934, Lopes (1934) published his monograph in which he mentioned *caapi* in the journal *Arquivos Brasileiros de Higiene Mental* , a Brazilian scientific publication that disseminated eugenicist ideas and was linked to the controversial Brazilian Mental Hygiene League ( Muñoz, 2015 ). Lopes’ research also demonstrates the prestige of eugenic notions from the viewpoint of researchers who focused on these plants. Costa and Faria (1936) cited his work and the “brilliance of his intelligence,” calling for the Brazilian scientific community and authorities to look at the therapeutic uses of *yagê* : “Countries with scant forest resources organize congresses to study their plants. Unlike them, we Brazilians, who hold the world’s greatest plant wealth, sleep without concern” (Costa, Faria, 1936, p.622).

Despite its potential for treating public health problems, therapeutic use by eugenicists raised a warning about the risks involved in scientific use of ayahuasca as a way of controlling bodies and minds. At that time, certain hospitals and insane asylums in Europe like the Prussian National Asylum and the Epileptics’ Hospital (both in Potsdam, Germany) were already conducting research with this substance on patients considered by eugenicists to be mentally inferior, as described by Miranda (2013) . Rudolf Wedel, who presented his work on *yagê* to the German Spagyric Medicine and Homeopathy Society, wrote a letter to the Brazilian homeopathic physician Alcides Nogueira da Silva, recommending he investigate the use of ayahuasca to treat epilepsy and “nervous diseases” (Costa, Faria, 1936).

The fact that ayahuasca was considered by eugenicist psychiatrists as a new treatment option for individuals thought to be less mentally fit also leads us to question, alongside the Brazilian historian Vanderlei Sebastião de Souza (2016) , the notion that the Latin American version of eugenics was “milder” than that seen in Europe and the United States. This author maintains that although the measures seen in countries in the northern hemisphere were based on extreme policies of racial segregation and control of human reproduction, the Brazilian eugenics movement also carried out campaigns with social repercussions, like projects to “whiten” the population and to blame Blacks and other individuals for the country’s civilizational “backwardness,” which they believed would be resolved by scientific development.

As mentioned earlier, even considering its potential for clinical applications, these researchers believed that ayahuasca required special caution because of its indigenous origin and even the scientific uses of this substance. Costa and Faria (1936 , p.397) called it a narcotic that could be put to other uses in the city:

Another narcotic! Is this revelation cause for joy or sadness? We believe we are not wrong in stating that given the current subversive situation in the world, our feelings are mixed. We are happy to know that chemistry will present medicine with yet another powerful therapeutic agent, and sad to foresee the possibility that its true use may be perverted.

Lopes (1934 , p.116) himself thought the vine could “cause psychiatric disorders” and affect the brain: “Various plants from the mysterious Amazon offer and exhibit extraordinary marvels for study. *Yagê* is one of these marvels so extraordinary that they could become harmful.” He also warned that its scientific and therapeutic uses and presence in cities could expand. He mentioned its indigenous uses for soothsaying, citing a passage in Lewin’s 1924 text *Phantastica* stating that the vine was thought to be associated with “personal happiness,” but related it to the “savages” and immorality from a Christian viewpoint, in our view defining the cosmovision prevalent when this substance was first scientifically utilized.

This is similar to the discussion on *Cannabis* spp.; the Brazilian historian Thamires Moreira (2019) reports medical recommendations of the therapeutic uses of *Cannabis indica* in the book of observations from the Hospital Nacional dos Alienados in Rio de Janeiro in the early twentieth century. But the use of this plant was moralized, since it was associated with Afro-Brazilian populations who used it for medicinal and religious purposes and to resist western moral and legal impositions. For this reason, authorities and researchers mobilized to include it in the Penal Code, which established penalties for charlatanism. This does not differ greatly from the current situation, even in discussions on the medicinal uses and chemical properties of the plant.

Costa and Faria (1936 , p.621) also viewed the 1929 launch of Cerebroil by the Leysin Laboratory in Paris; this compound contained yageina and was recommended for “cerebral insufficiency and reeducation,” nervous depression, memory loss, phobias, and “increased intensity of thoughts:” “And we thought charlatanism only existed in Brazil!” This is proof of the pharmaceutical industry’s interest in this psychoactive substance in the early twentieth century. At that time, Sandoz was already working to identify compounds that could be marketed to the population; this how LSD, which Albert Hofmann found in 1938, was first sold ( Delmanto, 2020 ; Strassman, 2019 ). More recently, authors like Beserra and Vieira (2020) have called attention to pharmaceutical manufacturers’ interest in psychoactive substances.

In the case of ayahuasca, we note that therapeutic usage requires reflection on specifically how this can take place: for example, via micro-dosing, since people are already selling such projects illegally in groups on the internet ( Lima, 2021 ), whether ayahuasca could be provided through the public health system, and whether this would involve regulatory approval processes (involving clinical trials, pricing etc.) like other medications. It also raises questions about who ayahuasca belongs to, as historical and cultural heritage (Assis, Rodrigues, 2017), and whether indigenous people should receive a portion of manufacturer profits, for example.

But for this to occur, pharmaceutical manufacturers will need to enter the fray about the historical criminalization of ayahuasca and other psychoactive substances as “drugs.” The Brazilian anthropologist Júlio Assis Simões (2008) explains that in common parlance, “drug” is used as a category to describe illicit psychoactive substances like marijuana, cocaine, and ecstasy whose use is considered a personal and public health problem associated with crime and violence, and therefore is the target of control and prevention policies. But there are some inconvenient details in this association between drugs and the abuse of illicit psychoactive substances, such as the pathological determination of drug addiction and view of drugs as a threat to society. This leads to the “war on drugs” waged by prohibionist policies that encourage stigmatization, naturalize illegality, implement repression, and lack intellectual discussion while also favoring organized crime, illegal commerce, and international trafficking.

While the religious and scientific uses of ayahuasca have been regulated in Brazil since 2010, it is not excused from this debate. Just a few months after this legislation was passed, the well-known Brazilian cartoonist Glauco and his son Raoni were murdered at an ayahuasca church in São Paulo by a member of the congregation who was later diagnosed with schizophrenia. Public outcry followed, leading the press, politicians, religious authorities, and part of the scientific community to question the uses of ayahuasca, which was presented as a drug ( Carvalho et al., 2016 ).

In this debate, plants like coca also have been historically criminalized. The Brazilian psychologist and anthropologist Ivan Farias Barreto (2013) mentions that coca leaf, which is used in its natural state by Andean peoples, is associated with narcotrafficking by westerners even though the plant contains only minute quantities of cocaine, and has been targeted by political schemes to ban its production and consumption. When considered within the same vague category of “drugs,” the history, uses, non-western classifications, and biodiversity of plants used by traditional peoples are generalized rather than allowing serious and non-biased discussions about each one individually. These are only some of the layers by which the present and past meet in history, and in which the psychedelic renaissance could in fact offer a new moment for reflection and discussions on psychoactive substances.

## Final considerations

Reports from explorers and travelers in the latter half of the nineteenth century and studies during the early decades of the twentieth century show that self-proclaimed “civilized” people were interested in the therapeutic properties of ayahuasca to treat “nervous diseases,” for example, and also as a “magical” plant that caused insightful dreams and revelatory visions. Notably, eugenicists were interested in this substance and utilized it in experiments on people considered risks to society, and the pharmaceutical industry also took interest. But the researchers themselves moralized the debate and encouraged criminalizing ayahuasca use by indigenous people as well as in the city, since the plant could “addict” westerners and even act as the “revenge” of exploited peoples.

This historical aspect casts light on the current political debate on ayahuasca with regard to various issues mentioned herein, such as the presence of an epistemological scenario that moralizes and criminalizes indigenous uses and colonizes classifications, reducing the species of plants and their chemical properties and meanings to western parameters; questioning the psychedelic renaissance, particularly the therapeutic potential of psychoactive substances as potential treatments for public health problems, while being careful to control bodies and minds; interest in ayahuasca by pharmaceutical manufacturers and the need to regulate these uses; and a debate on drugs that discusses prohibitionism itself and its effects on society, including researchers who have conducted studies in this area for years and assessments of public policies in the dialog.

Furthermore, ethnographic studies with different indigenous groupings could provide more information about the varietal diversity of plants used to prepare ayahuasca, and alongside western chemistry, the makeup of these plants according to the indigenous cosmovision. All of these points could guide a research agenda on ayahuasca and other psychoactive substances, particularly those used by traditional populations.

In this sense, a reading of the historical aspects of actor-network theory seems useful in planning by offering theoretical and methodological elements to consider the network and the actors, associations, controversies, layers, and temporalities that comprise them, including researchers in the history of science and indigenous history in this network. In this way, we were able to determine that the contradictions around ayahuasca are not recent, and we identified elements to construct a new history (linked to past experiences) that is not intended just for the western science that expresses interest in the therapeutic potential of this substance, but also for the indigenous people who have used it for millennia. Finally, even though ayahuasca has currently gained legitimacy in the public debate, learning about its past shows that even though psychoactive substances may be seen as potentially revolutionary in areas like psychiatry, long-standing misunderstandings in the political sphere must be confronted.

## NOTES

1 While we use the term ayahuasca (which is most commonly found in modern science) throughout this article, we shall also discuss this western classification.

2 For this and other citations from non-English sources, a free translation has been provided.

3 Reichel-Dolmadoff (1976) mentions 1851, but Costa & Faria (1936) state 1853; other dates have also been proposed for this event ( Schultes, 1986) .
